# Ensuring breastfeeding equity has promise to help equalize early childhood development^⁎⁎^

**DOI:** 10.1016/j.jped.2025.06.001

**Published:** 2025-06-27

**Authors:** Cecília Tomori

**Affiliations:** Johns Hopkins School of Nursing and Johns Hopkins Bloomberg School of Public Health, Baltimore, USA

Early childhood development (ECD) is the cornerstone of our collective future – with lifelong impacts for health, wellbeing, educational attainment and economic participation.^1^ This is why ECD is a major global priority, and its embedded throughout the Sustainable Development Goals.^2^ To help children achieve their full potential, ECD must be supported by multi-sectorial, structural measures that address poverty, malnutrition, environmental harms, and inequities in access to healthcare and education, among others.^1^^,^^3^ Today, however, globally we face inequities that continue to limit children’s development and disproportionately affect children living in low- and middle-income countries (LMICs).^4^ Breastfeeding has powerful, life-long effects on child health and development.^5^ In their innovative new study, Ford et al.^6^ examine the role of exclusive breastfeeding as a potential moderator of developmental outcomes in a cohort of children drawn from Southeast Brazil whose mothers had unequal patterns of educational attainment. ECD was measured at 12 months via an in-person assessment using the Bayley-III validated scales. Since maternal education is a well-established determinant of ECD,^7^ the authors hypothesized that exclusive breastfeeding would have a protective effect for those children whose mothers have lower educational attainment.

In concert with previous findings, the study found that children of mothers with higher educational attainment had more optimal early childhood development, as reflected by cognitive, language and a composite Bayley Global Scores. Similarly, in line with prior literature, EBF was also associated with higher cognitive, language, and Bayley Global Scores. Notably, when the authors carried out a moderation analysis, they found that children of those with fewer years of schooling (under 10 years and between 10 and 12 years) but who were EBF attained higher cognitive and global scores than those who were not EBF. A sensitivity analysis further found a medium effect size for the cognitive scores and a large effect size for the global scores among those with the fewest years of schooling (under 10 years).

These are important findings because they show that EBF has a protective impact for children whose mothers face educational disadvantages. This reinforces the importance of breastfeeding for developmental outcomes and for ensuring positive outcomes across the life course, especially for children who may face early disadvantages. EBF confers nutritional advantages as well as opportunities for mothers and infants to interact and facilitates responsive, nurturing care that leads to healthy development.^5^ Breastmilk is a living substance that exists within the context of the dynamic feedback loops of the breastfeeding relationship.^5^ Importantly, breastfeeding is a complex package of evolutionary adaptations that encompasses all of these biopsychosocial elements and many more.^5^ The study ultimately reinforces that breastfeeding is a matter of ensuring intergenerational health equity and scaling up support for it should be of central concern for governments.

The 2023 Lancet Breastfeeding Series documented that while most women want to breastfeed, they are unable to do so due to numerous structural and social barriers.^5^ Key among these are practices in the health system that fail to adequately support breastfeeding, workplaces that do not provide adequate leave and supportive environments for breastfeeding, and aggressive marketing tactics by the formula industry that undermine breastfeeding by misleading caregivers, target health professionals and shape the policy environment.^5^ Together, these barriers make it difficult to make informed decisions about infant feeding, initiate breastfeeding in a timely manner after birth, sustain exclusive breastfeeding for 6 months and to continue it with complementary foods for 2 years as recommended by the World Health Organization (WHO). Substantial positive changes have been achieved in Brazil over the last 20+ years in early initiation of breastfeeding, EBF at six months, and continued breastfeeding at 1 and 2 years.^8^ Yet, breastfeeding outcomes still fall considerably short of 2030 targets. Indeed, Brazil’s national EBF rate in 2019 was 45.8 %.^8^ Although the Southeast region of Brazil from where this cohort hails had higher EBF rates in 2019 at 49.1 %, it would still need to significantly increase these rates to meet 2030 goals of 70 %.^8^ In this cohort, which was born in 2022 and impacted by the COVID-19 pandemic, the authors note that EBF rates were lower at 37.92 % than the previously collected national and regional averages.

The authors note that a driver of these lower EBF rates may be economic pressures compounded by the pandemic, which forced those especially from lower socioeconomic groups to enter work environments that were less accommodating for breastfeeding.^9^ This highlights the importance of structural support in the workplace for breastfeeding, particularly for those working in the informal sector and low-wage jobs.^5^ Greater educational attainment may mean better economic opportunities and more flexibility to find a workplace that is supportive of breastfeeding.^10^ Although we do not have data on the work environments of the participants, those with lower educational attainment had lower EBF rates (28.6 % for mothers with < 10 yrs, 36.5 % for those 10–12 yrs and 43.5 % for those with >12 years of education, respectively). Moreover, while women in Brazil have made significant progress in educational attainment, their earnings are still significantly lower across all levels.^11^ The Equal Pay Law enacted in 2023 to address the gender wage gap may help to address these inequities.^12^

The health system is another major driver of these lower EBF rates.^5^ The authors mention the importance of the implementation BFHI in Brazil. A key challenge has been the stagnating number of BFHI hospitals, and uneven implementation of the 10 steps that are part of the BFHI.^13^ Moreover, breastfeeding supportive hospital practices and direct support services were frequently disrupted during the COVID-19 pandemic,^14^^,^^15^ which may have also had an influence for this cohort. An area of further study would be to examine ECD in relation to the trajectory of breastfeeding experiences and early breastfeeding outcomes, taking into account BFHI practices and COVID-19 policies, and the socioeconomic status and educational attainment of mothers.

Although Brazil has strong protections for breastfeeding, and has endorsed the International Code of Marketing of Breast-Milk Substitutes (the Code), it has also witnessed significant rise in commercial milk formula consumption in the past two decades.^16^ This is due to persistent corporate political activities and aggressive marketing efforts that target families, health professionals and their organizations, and government policymakers.^16^ These tactics are pervasive globally and constitute the commercial milk formula playbook that is deployed to generate profits at a high cost for public health and health equity.^17^ Another avenue for further investigation is the exposure and impact of these marketing practices by educational status with downstream impacts for ECD.

For most major drivers of EBF inequities are well documented along the lines of maternal socioeconomic status, and educational attainment is a key component of SES. So, while EBF can help buffer the relationship of educational attainment and ECD, the ability to EBF for 6 months may itself be constrained by these structural barriers. In agreement with the authors, longitudinal studies will be needed to untangle these complex relationships with confounders and to determine causality. Further governmental investment and multi-sectorial collaboration are necessary to help remove these structural barriers to create enabling environments for breastfeeding that are equitable across the social spectrum.^5^ Women should have the opportunity to fully participate in education, earn equitable pay, and be supported in their breastfeeding journeys in the health system, workplace, and beyond–which in turn impacts their children’s development, health and wellbeing. Together, these findings reinforce that governmental support for gender equity and breastfeeding matters for mothers and their children–and ultimately all families and communities across generations.

## Conflicts of interest

The authors declare no conflicts of interest.
